# Zoonotic Diseases: Etiology, Impact, and Control

**DOI:** 10.3390/microorganisms8091405

**Published:** 2020-09-12

**Authors:** Md. Tanvir Rahman, Md. Abdus Sobur, Md. Saiful Islam, Samina Ievy, Md. Jannat Hossain, Mohamed E. El Zowalaty, AMM Taufiquer Rahman, Hossam M. Ashour

**Affiliations:** 1 Department of Microbiology and Hygiene, Faculty of Veterinary Science, Bangladesh Agricultural University, Mymensingh 2202, Bangladesh; soburvetbau@gmail.com (M.A.S.); dvm41257@bau.edu.bd (M.S.I.); v.samina@gmail.com (S.I.); md.jannat.hossain@gmail.com (M.J.H.); 2 Department of Clinical Sciences, College of Medicine, University of Sharjah, Sharjah 27272, UAE; elzow005@gmail.com; 3 Zoonosis Science Center, Department of Medical Biochemistry and Microbiology, Uppsala University, SE 75123 Uppsala, Sweden; 4 Adhunik Sadar Hospital, Naogaon 6500, Bangladesh; drtaufiqurahman@gmail.com; 5 Department of Integrative Biology, College of Arts and Sciences, University of South Florida, St. Petersburg, FL 33701, USA; 6 Department of Microbiology and Immunology, Faculty of Pharmacy, Cairo University, Cairo 11562, Egypt

**Keywords:** zoonosis, pathogens, viruses, bacteria, fungi, animal, SARS-CoV-2, COVID-19, one health, prevention

## Abstract

Most humans are in contact with animals in a way or another. A zoonotic disease is a disease or infection that can be transmitted naturally from vertebrate animals to humans or from humans to vertebrate animals. More than 60% of human pathogens are zoonotic in origin. This includes a wide variety of bacteria, viruses, fungi, protozoa, parasites, and other pathogens. Factors such as climate change, urbanization, animal migration and trade, travel and tourism, vector biology, anthropogenic factors, and natural factors have greatly influenced the emergence, re-emergence, distribution, and patterns of zoonoses. As time goes on, there are more emerging and re-emerging zoonotic diseases. In this review, we reviewed the etiology of major zoonotic diseases, their impact on human health, and control measures for better management. We also highlighted COVID-19, a newly emerging zoonotic disease of likely bat origin that has affected millions of humans along with devastating global consequences. The implementation of One Health measures is highly recommended for the effective prevention and control of possible zoonosis.

## 1. Introduction

Humans, animals, and the environment play a significant role in the emergence and transmission of different infectious diseases [1]. Most of the infectious diseases affecting humans are of animal origin. The “Asia Pacific strategy for emerging diseases: 2010” report estimated that around 60% of the emerging human infections are zoonotic in nature and among these pathogens more than 70% originated from wildlife species [2]. The newly emerged diseases in humans in recent decades were of animal origin and were directly associated with animal origin foods [3].

The term “Zoonoses” is derived from the Greek word “Zoon”, which means animal, and “nosos”, which means illness. According to the World Health Organization (WHO), any disease or infection that is naturally transmissible from vertebrate animals to humans or from humans to animals is classified as a zoonosis [4]. Among the human pathogens, about 61% are zoonotic in nature [5].

Zoonoses is a great public health concern and a direct human health hazard that may even lead to death. Across the globe, the 13 most common zoonoses were most impactful on poor livestock workers in low- and middle-income countries and have caused an estimated 2.4 billion cases of illness and 2.7 million deaths in humans per year in addition to their negative effect on human health [6]. Most of these diseases affect animal health and decrease livestock production [6].

## 2. Classification of Zoonoses

Zoonotic diseases are caused by a wide range of pathogens. Based on etiology, zoonoses are classified into bacterial zoonoses (such as anthrax, salmonellosis, tuberculosis, Lyme disease, brucellosis, and plague), viral zoonoses (such as rabies, acquired immune deficiency syndrome- AIDS, Ebola, and avian influenza), parasitic zoonoses (such as trichinosis, toxoplasmosis, trematodosis, giardiasis, malaria, and echinococcosis), fungal zoonoses (such as ring worm), rickettsial zoonoses (Q-fever), chlamydial zoonoses (psittacosis), mycoplasma zoonoses (*Mycoplasma pneumoniae* infection), protozoal zoonoses, and diseases caused by acellular non-viral pathogenic agents (such as transmissible spongiform encephalopathies and mad cow disease) [7]. Table 1 lists the major zoonotic diseases with their etiological agents, animal host, and major symptoms.

The older classification of zoonoses includes the terms anthropozoonoses, zooanthroponoses, amphixenoses, and euzoonoses [8]. Anthropozoonoses are animal diseases that can be transmitted to humans, such as rabies. Zooanthroponoses refers to those diseases that are transmitted to animals from humans such as tuberculosis in cat and monkey. Amphizoonoses are those diseases that can be transmitted in any direction (from human to animal and from animal to human) such as staphylococcal infection. For some parasitic diseases, humans act as the obligatory host. These parasitic diseases are known as Euzoonoses such as *Taenia solium* and *Taenia saginata* infections.

Both Gram-negative and Gram-positive bacteria are capable of inducing zoonoses. Based on etiology, bacteria account for most zoonotic diseases. It has been estimated that among the zoonotic pathogens originating from bovine origins, about 42% are of bacterial origin, 22% viral, 29% parasitic, 5% fungal, and 2% prion origin [9]. Similarly, both DNA and RNA viruses are known to be responsible for zoonoses; however, compared to DNA, the RNA viruses are more frequently linked with zoonoses [10].

Pathogens can be transmitted to humans directly or indirectly from animals. Diseases that are transmitted directly to humans from animals through media such as air are known as direct zoonoses [11]. One classical example of direct zoonoses is avian influenza, which is a viral disease that spreads from animals to humans through droplets or fomites. Infected animals can also directly transfer pathogens to susceptible humans by bites such as in the case of rabies, which is one of the deadliest zoonotic diseases. It is caused by a rabies virus that belongs to *Rhabdoviridae*. When a rabid animal (dog, bat, monkey, skunk, raccoon, or fox) bites a human, the virus directly enters the human body through saliva. Similarly, pathogens can be transmitted to human via vectors (Dengue fever). Arthropods like mosquitoes and ticks are often considered to be the only vectors; however, any animal that has the potential to transmit pathogens to human can be considered a vector [12].

Depending on the ecosystem in which pathogens circulate, zoonotic diseases are classified into several categories. For example, some zoonoses are classified into synanthropic zoonoses and exoanthropic zoonoses. Synanthropic zoonoses have an urban (domestic) cycle in domestic and synanthropic animals such as with urban rabies and zoonotic ringworm. Exoanthropic zoonoses are usually accompanied by a sylvatic (feral and wild) cycle in natural foci outside human habitats such as with arboviroses, wildlife rabies, and Lyme disease [13]. However, some zoonoses can also circulate in both urban and natural cycles such as yellow fever, Chagas disease, and dengue fever. In addition, there are some other zoonotic diseases that can be transmitted by arthropods, food, rodents, and some are waterborne [14].

Many zoonotic pathogens are able to replicate in and survive on dead organic materials like saprophytes and the diseases caused by these agents are known as sapronoses. Examples of sapronoses include fungal diseases (such as coccidioidomycosis, histoplasmosis, and aspergillosis) and bacterial diseases (such as legionellosis) [15]. The term “saprozoonoses,” is defined by the WHO expert committee on zoonoses as pathogens that have a vertebrate host as well as a non-animal reservoir or developmental site (soil, plants, and organic matter) [16]. In many cases, disease transmission may require more than one vertebrate host such as with human taeniasis. These types of zoonoses are known as cyclozoooses. Zoonoses in which both vertebrate and invertebrate hosts are involved are known as metazoonoses such as with arbovirus infection.

Most zoonotic diseases are transmitted to humans from animals. Some reports suggested that animals can also get infected from humans [17,18,19,20]. Such diseases are known as reverse zoonoses. Examples of such pathogens include methicillin-resistant *Staphylococcus aureus* (MRSA), *Campylobacter* spp., *Salmonella enterica* Serovar Typhimurium, influenza A virus, *Cryptosporidium parvum*, *Ascaris lumbricoides*, and *Giardia duodenalis*. In addition, zoonotic diseases caused by pathogens that are occasionally transmitted to animals from humans and then back from animals to humans are referred to as reverse zoonoses (Table 2).

## 3. Zoonoses of Domestic Animals

Domestic animals play a significant role in the transmission of various diseases to humans and in many cases, they work as amplifiers of pathogens emerging from wild animals [22]. The positive association between domestic animals and humans in influencing pathogen diversity was first hypothesized long time ago [23]. About 60% human infectious diseases come from vertebrate animals [5,24]. Direct human contact with animals has expanded with the introduction of domestication of different vertebrate animals [25]. The possible transmission patterns of zoonotic bacteria, virus, parasites, or fungi are via direct contact, ingestion, inhalation, through conjunctiva, or biting [24].

Cattle, sheep, goats, dogs, cats, horses, pigs, and other domestic animals act as reservoirs of pathogens of domestic zoonoses and can transmit the diseases to humans [26]. Pathogens can be transmitted through direct contact or animal origin foods. Examples of zoonotic diseases that can be transmitted to humans from domestic animals include anthrax, rabies, tuberculosis, brucellosis, campylobacteriosis, leptospirosis, toxoplasmosis, balantidiasis, ancylostomiasis, toxocariasis, listeriosis, bovine pustular stomatitis, rotavirus infection, and Q fever [10,26,27].

Of these zoonotic diseases transmitted by domestic animals, anthrax caused by *Bacillus anthracis* poses a significant public health importance. *B. anthracis* is soil-borne bacteria with the capability to produce spores; thus, allowing them to survive in the environment for a very long time. Anthrax can be transmitted to humans through close contact with infected animals (such as cattle and goat) or their products (such as meat, skin, hides, or even bones). [28]. Human to human transmission exists, but it is very rare. Every year, about 2,000–20,000 humans are affected by anthrax cases globally [28]. People from India, Bangladesh, Pakistan, United States, Zimbabwe, Iran, Iraq, South Africa, Turkey are occasionally affected [28]. In humans, it can develop malignant pustule, gastroenteritis, pneumonitis; conversely sudden death with some systemic lesions can occur in animals. Mortality can be 25–65% in intestinal anthrax; however, it may rise up to 100% in pulmonary anthrax [29]. Developing countries whose economy usually depends on agriculture are still facing hazardous effects due to anthrax.

Among the bovine zoonoses having serious public health significance, tuberculosis is the most important zoonotic disease. The disease has been significantly a cause for severe economic loss in animal production. It is caused by *Mycobacterium bovis*, *M. tuberculosis,* or rarely *M. caprae* [30,31,32]. *Mycobacterium* are acid-fast soil saprophytes characterized by the presence of mycolic acid in their cell wall. They are also facultative intracellular pathogens. Though bovine tuberculosis has been greatly eliminated from developed countries, other parts of the globe are still facing serious zoonotic effects. Human tuberculosis is the second most common cause of death after AIDS. About 5–10% of all human tuberculosis has been caused by *M. bovis* (25% of the patients were children). About 53% of all cases showed that the favorable site of tuberculosis is the extra-pulmonary tract [26]. Most humans are affected with tuberculosis by handling or milking unpasteurized contaminated milk or via aerosols from coughing of infected animals [33]. Importantly, *M. bovis* infection can also happen in the urogenital system of humans and can impact animals through the respiratory secretions from humans acting as reverse zoonoses [34]. However, direct contact of infected animals with humans such as farm workers, veterinarians, abattoir workers, or village people can pose a significant risk.

Brucellosis is one of the most common bacterial zoonotic diseases causing over 500,000 human cases throughout the world every year [35]. The disease is classified as a forgotten neglected zoonosis as per the WHO [36]. Among the twelve species of the genus *Brucella, Brucella melitensis*, *B. abortus, B. suis*, and *B. canis* are zoonotic in nature. The common transmission pattern of brucellosis to human occurs through the consumption of unpasteurized milk or milk products, though the human–human transmission is rare. Transmission through the inhalation of aerosols and contact with secretions has also been reported [37]. In humans, brucellosis mainly causes influenza-like infections, pneumonia, and other complications including meningitis, endocarditis, septicemia, serious weakness, pain in muscle and joints, extreme headache, fever, and night sweats. In animals, brucellosis causes abortion, lameness, abscess, reduction in milk production, and decrease in survival chances of newborns [10,38]. Dairy farm workers, caretakers, abattoir workers, veterinarians, and village people are at high risk for brucellosis infection.

Rabies is one of the deadliest zoonotic disease caused by rabies virus, which belongs to *Rhabdoviridae.* Every year about 30,000–70,000 human deaths occur throughout the globe [39]. Though dogs are the main carriers of rabies virus, other wild animals including cats and jackals also act as carriers for the transmission of rabies virus. In developing countries, humans are affected by rabies through biting because of the stray dog problem [40]. In developed countries, bats, foxes, and other wild animals are responsible for the transmission of rabies [40]. Severity and gravity of wound, its anatomical location, and viral load can influence the incubation period of rabies, which may vary from four days to several years [41,42,43]. Furious or classical or encephalitic form and paralytic or dumb form are the diversified clinical characteristics of rabies, though they are usually dominated by viral tropisms and neural sites and spread, volatile immune response or other potential mechanisms [43,44,45,46,47,48]. The most common symptoms of the disease include excitation, solicitude, anxiety, bewilderment, hallucination, and hydrophobia [27].

## 4. Zoonoses of Pets, Companion Animals, and Birds

About 14–62% of pet owners allow their pets to their bedrooms, which could enhance the emergence of zoonoses [49]. Companion and pet animals have increased over the past several decades, but they are also a comprehensive source of disease-producing agents. The increased popularity of pets and companion animals has put human health at risk due to the possible spread of infections. In many houses nowadays, pets of exotic species are kept along with common pets. Therefore, huge people are at risk of acquiring new zoonotic disease from pets, companion animals, and exotic birds and animals.

A variety of infectious diseases (viral, bacterial, parasitic, and fungal) are associated with pets and companion animals [50]. The zoonotic diseases frequently associated with pets and companion animal include brucellosis, campylobacteriosis, chlamydiosis, catch scratch fever (*Bartonella henselae*), ehrlichiosis, giardiasis, hantavirus, hookworms, influenza, rabies, Lyme disease, rocky mountain spotted fever, leptospirosis, monkey pox, pasteurellosis, Q fever, plague, roundworms, salmonellosis, staphylococcosis (MRSA), streptococcosis, toxoplasmosis, and tularemia [50,51,52]. Many types of zoonoses such as salmonellosis, staphylococcosis, and rabies are found in a wide range of pets and companion animals.

Nowadays, birds like canaries, finches, sparrows, parrots, parakeets, budgerigars are very common in the developed and developing countries [53]. Like pet animals, these game and ornamental birds are also potential transmitters of zoonotic diseases like *Coxiella burnetii*, *Coxiella psittaci*, *Salmonella* spp., *Listeria monocytogenes*, *Erysipelothrix rhusiopathiae*, *Mycobacterium* spp., Lyme disease, and transmitters of different viruses like fowl pox virus and Newcastle disease virus [54]. Many of these pathogens are potentially enough to cause serious diseases in human such as salmonellosis, chlamydiosis, and avian influenza A H5N1 [53]. In addition, there are wide varieties of other bacterial zoonoses in game and ornamental birds including *Pasteurella* spp., *Klebsiella* spp., *Yersinia* spp., *Pseudomonas* spp., *Staphylococcus aureus* and *E. coli* [55,56,57]. In fact, there is evidence of *Escherichia coli* O157:H7 (enterohaemorrhagic) transmission to human through food of animals that originally came from wild passerines (such as European starlings) [58].

Transmission of pathogens from these animals occurs through direct or indirect contact. The transmission can take place at home, outside, pet shops, hospitals, or other places. In many cases transmission also takes place when these animals and birds are brought to shows and competitions [59,60]. Usually, animal bites or scratches are routes through which humans get the infection such as pasteurellosis and cat scratch disease [61].

It is noteworthy that the most common dog-associated zoonotic disease is rabies is caused by rabies virus that kills tens of thousands of people every year [62]. Similarly, pet-associated MRSA is a serious health problem for human across the globe [63].

One of the important zoonoses associated with pet is cat-scratch disease. The etiological agent of the disease is *Bartonella henselae*. Cat-scratch disease is a common infectious disease and is usually benign. Cat-to-cat transmission of the disease takes place horizontally, but humans occasionally gets the infection by arthropod vectors like fleas and ticks. In addition, the most frequent transmission patterns in human include cat licking of open wounds of a person, or bites and scratches causing wounds. The incubation period of the disease varies from 3 to 14 days. Several lesions like swelling and redness with raised, round areas may appear, and pus may also form at the site of infection. Moreover, the lymph nodes near the bitten or scratched area or on the neck region are usually swollen [64]. Rearing pets with good hygiene practices as well as routine vaccination and medical checkup is needed to ensure their freedom from these kinds of zoonotic infections.

## 5. Zoonoses of Fish and Aquatic Environments

Many microorganisms with zoonotic significance have been isolated from fish [65]. Fish-associated zoonotic pathogens are mainly bacteria. Often, fish unsusceptible to these infections are capable to cause serious sickness in humans. However, these opportunistic fish-borne bacterial infections are limited. Fish can get these pathogens from the aquatic environment where they remain as an indigenous part. In addition, aquatic environments may get contamination from agricultural activities, human and animal excreta, garbage from households, and wild animals. These zoonotic infections may be transmitted to humans through the non-hygienic handling of aquatic animals and/or their products. Consumption of raw or improperly cooked aquatic products may also transmit foodborne infections to humans. Among the zoonotic pathogens isolated from fish, *Aeromonas hydrophila*, *E. coli*, *Yersinia* spp., *Brucella* spp., *Shigella* spp., *Salmonella* spp., *Streptococcus iniae*, *Clostridium botulinum*, *Klebsiella* spp., and *Edwardsiella tarda* are important [66,67].

Several *Vibrio* species, at least 12, are often known to be potential for fish-associated zoonoses [68]. Among them *Vibrio cholerae*, *V. parahaemolyticus*, *V. vulnificus*, *V. damsela* are mostly involved in human illness [69,70]. Eating contaminated raw or undercooked seafood is the major way through which humans get these *Vibrio* infections, which can cause serious symptoms such as diarrhea, vomiting, and dehydration [71]. These fish-associated pathogens may be transmitted to humans through accidental ingestion of contaminated fish products (mostly contaminated with fish fecal materials) or directly though broken, wounded skin. In most of the cases, these fish pathogens are endogenous in origin. Cross-contamination with zoonotic pathogens like *Listeria monocytogenes* in ready-to-eat fish products has recently been reported [72].

In humans, *Mycobacterium tuberculosis* cause TB. However, fish are susceptible to non-tuberculous mycobacterial infections. The infections are commonly associated with display aquaria and occasionally with commercial aquaculture systems. They can also be transmitted to humans during aquaculture practice in farm and handling of ornamental fish in aquarium and equipment [73]. *M. chelonae*, *M. marinum*, and *M. fortuitum* are main concerns in aquaculture and fish-related businesses. Among these, *M. marinum* is a well-known zoonotic pathogen. In fish, *M. marinum* infection is chronic in nature and is characterized by granuloma formation involving different organs and tissues, discoloration and loss of scales, loss of appetite, and apathy [74]. Similarly, in humans too, it causes granulomatous inflammation and nodule formation in skin, subcutaneous tissues, and tendons [75,76] and they are referred to as “fish tank granuloma”, “swimming pool granuloma”, “fish TB”, “fish handlers”, or “fish fancier’s disease” [77].

*Erysipelothrix rhusiopathiae* is a fish-borne pathogen that causes systemic skin diseases in marine mammals [78]. It is a Gram-positive pathogen but there is no reported disease in fish that is caused by this bacterium [79]. Similar to other fish-borne pathogens, human and non-human animals are exposed to this bacterium through direct contact with cutaneous wounds on fish [65]. *E. rhusiopathiae* can cause diseases in humans (known as “erysipeloid”) and animals (known as “erysipelas”) [80]. Fisheries workers are directly vulnerable to the transmission of *E. rhusiopathiae* during the handling and processing of live and dead fish, which is the reason that the disease is also referred as fish-handler’s disease [81]. The disease is also referred to as “fish rose” due to its symptoms, which include purple or red discoloration of the skin [81,82]. Although *E. rhusiopathiae* commonly causes localized infections, such as painful self-limiting cellulitis and edema, and systemic infection is rare in human, it can still cause serious endocarditis [81,83,84]. Among animals, pigs are the most vulnerable to *E. rhusiopathiae* and develops a disease entitled as “diamond skin disease” [80]. However, as the bacterium can survive in frozen conditions [85], fish handlers must take precautions during handling and processing of both fresh and frozen fish (such as wearing gloves or protective sleeves) and during cleaning the fish tanks or housing areas.

*Lactococcus garvieae* is an important fish-borne pathogen affecting a wide range of wild fish species (both marine and fresh water fish), giant prawns from fresh water, and wild marine mammals [86]. This bacterium causes severe hyperacute hemorrhagic septicemia (known as lactococcosis) in cultured warm-water fish with high mortality rates and an ultimate ominous impact on aquaculture industry [80,87,88]. *L. garvieae* has been isolated from ruminants (with subclinical mastitis), cow’s milk, beef meat, goat cheese; and poultry meat, and pork (from pigs with pneumonia) [86,89]. *L. garvieae* is an emerging zoonotic fish-borne pathogen that causes endocarditis, bacteremia, peritonitis and/or bacterioascites, espondilodiscitis, diskospondylitis, hip prosthetic infection, acalculous cholecystitis, and meningitis in human [86,90,91]. The transmission patterns of *L. garvieae* from fish to humans include direct contact with the contaminated raw fish during handling and processing and the consumption of contaminated raw fish and seafood [86].

*Nocardia* spp. affect fish such as teleost, shellfish, rainbow trout, blueback salmon, and pacific oyster causing nocardiosis, which is a lethal granulomatous disease developed in the skin, muscle, and inner tissues [92]. Examples include *Nocardia asteroides*, *N. seriolae*, *N. salmonicida*, and *N. crassostreae*, which are all associated with fish and aquatic environments. The primary route of transmission is via wounds [93]. *Nocardia* spp. have also been reported in humans, ruminants (cattle and goats), cats, and marine mammals [94,95]. Among different *Nocardia* spp., *N. farcinica*, *N. brasiliensis*, and *N. otitidiscaviarum* are closely associated with human infections whereas *N. asteroides* is occasionally associated with human infections [80]. Generally, immunosuppressed patients are more vulnerable to nocardiosis. The disease is characterized by skin infections, pneumonia, and ulcers [96]. It is important to mention that there is a scarcity of data on genetic or epidemiological linkages between piscine and human nocardiosis [80].

## 6. Zoonoses Associated with Food-Borne Pathogens

Food acts as an important medium to transmit pathogens—known as food-borne pathogens—which usually cause diarrheal diseases. Many food-borne illnesses are caused by zoonotic pathogens. Food-borne pathogens can cause significant morbidity and mortality in adult and pediatric populations. Mortality, which impacts millions, is often associated with diarrheal diseases caused by contaminated food and drinking water [97]. It is estimated that about 600 million (1 in 10 persons around the world) people annually consume contaminated food and water. Among these affected people, 420,000 people including 125,000 children die [98]. Risk factors influencing food-borne zoonoses include handling and slaughtering of animals without appropriate precautions, and consuming undercooked animal-based food.

Common food-borne zoonotic pathogens include *Salmonella* spp. (*Salmonella enterica* serovar Enteritidis), *Campylobacter* spp., Shiga toxin-producing *Escherichia coli* (STEC), and hepatitis E virus. More than 90% of bacteria-triggered food-borne illnesses are caused by *Salmonella* spp. and *Campylobacter* spp. [99]. All domestic livestock, including poultry, can act as a reservoir for bacteria causing food-borne illness [100,101,102].

STEC is also known as verodoxin (Verocytotoxin)-producing *E. coli*. They can be transmitted from contaminated foods to human by direct contact [103]. *Escherichia coli* O157:H7 serotype of STEC was recorded as a major cause of food-borne zoonotic illness in 1980s and 1990s. Toxins produced from STEC strains can cause serious illness in humans including gastrointestinal symptoms, kidney failure, and bloody diarrhea [104,105]. In serious cases, STEC can induce severe complications such as hemolytic uremic syndrome (HUS), which can have fatal consequences. STEC infections can cause severe illness with high mortality in both young and elderly populations [99].

In addition, *Brucella* spp., *Listeria* spp., *Clostridium* spp., BSE, norovirus, calicivirus, and other hepatitis viruses, mainly found in intestines of animals, can be transmitted through contaminated food materials. There are several risk factors influencing the occurrence of food-borne zoonotic diseases such as globalization of farm animals and their meat market, consuming uncooked or undercooked food from wildlife, an increasing number of immunocompromised patients, and lack of adequate awareness regarding proper hygiene and good sanitation.

## 7. Potential Zoonoses Transmitted by Edible Insects

Edible insects are under-utilized food sources with a usually high nutritional content. They are becoming more popular due to the increased demand for digestible, palatable, and nourishing food products [106,107]. Insects consumed by human include beetles, caterpillars, ants, bees, wasps, grasshoppers, locusts, true bugs, dragonflies, termites, flies, cockroaches, and spiders. About two billion people use some of these insects as food sources throughout the world, especially in Asia, South America, and Africa [108].

Many of these edible insects pose health risks to humans through causing allergies and other disease conditions [109]. For example, edible insects such as caterpillars can provoke allergic reactions in children, which can include the symptoms of drooling, difficulty in breathing and swallowing, pain, and generalized urticaria. In addition, consuming mopane caterpillars and silkworm pupa has been shown to induce anaphylactic reactions in humans [110]. Moreover, edible insects, such as beetles can harbor metabolic steroids that may trigger hyper-fertility, retardation of growth, jaundice, edema, and liver cancer [111]. Lepidoptera and Coleoptera contain cyanogenic substances that can cause enzyme inhibition [111]. Longhorn beetles contain toluene; a nervous system depressant that can negatively impact brain and kidneys [111].

Edible insects can carry different infectious pathogens and parasites. Pathogens have been extracted from edible insects such as *Campylobacter* spp. from darkling beetles or buffalo worms (*Alphitobius diaperinus*); Enterobacteriaceae from *Tenebrio molitor, Acheta domesticus,* and *Brachytrupes* spp.; and *Micrococcus* spp., *Lactobacillus* spp., and *Staphylococcus* spp. from *Zoophobas morio, Tenebrio molitor, Galleria mellonella*, and *Acheta domesticus* [109,112]. In addition, edible insects can act as a critical vector for the zoonotic food-borne pathogens *Salmonella* spp. and *E. coli* [113,114,115,116]. *Salmonella*, detectable in beetles, are vastly distributed in and around livestock areas posing serious threat to animals [110].

Parasitic infections have also been reported from edible insect consumption. For example, *Gongylonema pulchrum* and *Dicrocoelium dendriticum* infections having zoonotic potential are transmitted to human via ingestion of ants [117]. Edible insects, such as beetles, cockroaches, and flies, could also harbor *Isospora* spp., *Giardia lamblia, Balantidium* spp., *Toxoplasma* spp., *Entamoeba* spp., *Pharyngodon* spp., *Spirura infundibuliformis*, and *Sarcocystis* spp. [107,118].

## 8. Emerging and Re-Emerging Zoonoses

Emerging zoonosis is a zoonosis that is newly recognized, newly evolved, or has occurred previously but shows an increase in incidence or expansion in geographical, host or vector range [119]. At least 250 zoonoses were listed as emerging and re-emerging zoonotic diseases during the last 70 years. These diseases have been spread rapidly throughout the world with increasing incidence along with geographical range [6]. Human are affected due to close contact with animals which act as reservoirs for emerging and re-emerging zoonotic diseases [120].

Increased human-animal contact or interaction resulting from changes in human and animal behavior, habitat, ecology, vector biology, pathogen adaptability, change in farm practices, livestock production systems, food safety, urbanization, deforestation, and climate change are among the triggering factors for emergence of zoonotic diseases [121]. Wildlife can act as a source or a reservoir for emerging and remerging zoonotic disease-pathogens [122].

Emerging and re-emerging diseases have significant impacts, not only on public health, but also on socio-economic issues around the globe [123,124,125,126]. Among 175 reported emerging diseases, 132 diseases are considered to be emerging zoonotic diseases [5]. Another report estimated that about 60.3% of the emerging diseases can be categorized under zoonoses. Among them, 71.8% originated from wildlife [127].

Examples of major emerging zoonoses include avian influenza, bovine spongiform encephalopathy (BSE), feline cowpox, rotavirus infection, norovirus infection, Ebola, hantavirus infection, West Nile fever, canine leptospirosis, MRSA infection, cat scratch disease, severe fever with thrombocytopenia syndrome (SFTS), Middle East respiratory syndrome (MERS), severe acute respiratory syndrome (SARS), and the most recent coronavirus disease 2019 (COVID-19) [61,122,128]. On the other hand, rabies, brucellosis, Japanese encephalitis, tuberculosis (*M. bovis*), and *Schistosoma japonica* infection are considered to be re-emerging zoonoses in many parts of the world.

SFTS, caused by a bunyavirus member of the family *Bunyaviridae*, is a life-threatening disease that is characterized by the sudden onset of fever, thrombocytopenia, and leukopenia [129]. The vector of this disease is a tick named Ixodid (*Haemaphysalis longicornis*). Symptoms of SFTS include mild or severe febrile illness. The signs and symptoms are almost similar to those of hemorrhagic fever [130]. In severe cases, multi-organ failure occurs leading to the death of 6–30% of the patients [131]. The disease was first reported in May 2007 in central and northeast China [132]. People working in the mountain are more susceptible to the disease. In addition, people more than 50 years old are considered to be high-risk with higher rates of morbidity and mortality [133].

The disease can be transmitted by direct contact with animals and/or through contact vectors [134]. In endemic areas, animals carry antibodies that are specific to severe fever with thrombocytopenia syndrome (SETS) virus antigens [134]. The disease is usually transmitted horizontally. A similar type of the bunyavirus-associated disease, with SFTS-like clinical signs, was later detected in USA, Japan, and South Korea. These observations have caused the disease to be regarded as an emerging zoonotic disease in different parts of the world [135,136,137].

Middle East Respiratory Syndrome (MERS), which is transmitted to humans from camels, is an emerging viral zoonotic disease. MERS first emerged in Saudi Arabia in 2012 [138]. The disease is caused by a coronavirus known as the MERS coronavirus (MERS-CoV). MERS infections can cause significant mortality rates and deleterious public health impacts [139,140]. The human fatality rate from MERS is around 30–35% [141], while the disease is asymptomatic in infected dromedaries. In rare cases, infected dromedaries may develop mild respiratory symptoms [142]. MERS-CoV was shown to develop severe infections in the lower respiratory tract [143].

### 8.1. Wild Animals and Re-Emerging Zoonoses

Wild animals are intricately connected with humans, domesticated animals, and environmental components, and thereby directly contribute to the transmission and maintenance of different infectious diseases [144]. Globalization, habitat destruction, climatic change, and loss of species and biodiversity are disrupting the ecological relations among the one-health components, which ultimately triggers the emergence of zoonotic pathogens and causes alterations in their transmission patterns [145,146,147]. Pathogens carried by wild animals can impact human health and animal health, reduce agricultural production, and generate disturbance in wildlife [148].

Wild animals such as mammals, reptiles, birds, fish, and amphibians act as a reservoir of zoonotic pathogens with the potential of transmission to humans or other animal hosts. The involvement of wild animals in the epidemiology and transmission of zoonotic diseases is alarming. The transmission patterns of wildlife zoonoses are influenced by the nature of pathogen involved and climatic parameters such as temperature, humidity, and rain fall [122]. The emergence and re-emergence of these pathogens is dependent on their transmission patterns among wild animals, domestic animals, and humans. Factors impacting these processes include (1) rapid increase of the human population; (2) ease of local and global travel; (3) increased human exposure to animals and animal products; (4) wildlife farming; (5) hunting, handling, and transporting wildlife (including carcasses) with limited precautions; (6) consumption of wild meat (such as bush meat); and (7) differences in agricultural practices [123,148,149].

In emerging and re-emerging diseases, there is significant transmission of pathogens to humans from wild animals. Human infections are usually developed from wild animals via direct contact or vector-mediated sources (such as in case of rabies and/or lyssaviruses, hantaviruses, Nipah virus, West Nile virus, and causative agents of leptospirosis and ehrlichiosis). The spread of infections largely depends on human–human transmission such as with HIV, Ebola virus, and coronaviruses [148].

**Figure 1:**
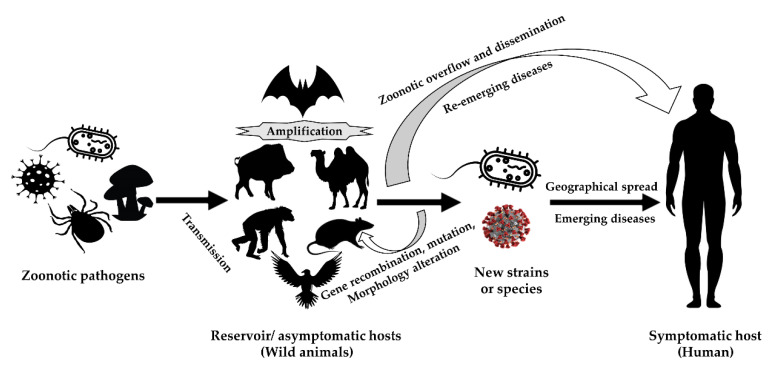
The involvement of the wild animals in the transmission and amplification of etiological agents of emerging and re-emerging zoonoses (modified with permission from [150]).

Figure 1 represents the role of wild animals in the transmission, amplification, and zoonotic overflow of causal agents of emerging and re-emerging zoonoses. In many cases, the wild animals act as reservoirs of causal agents that can stay dormant for long periods until triggered. The presence of agents in unusual hosts can sometimes increase the likelihood of errors in the process of RNA replication which can lead to mutations. This can lead to the generation of newer strains or species that can be more virulent, and/or more resistant to antibiotics. Consequences can include increased transmission rates in susceptible populations [150,151,152,153,154].

### 8.2. Zoonotic Coronaviruses

**Figure 2:**
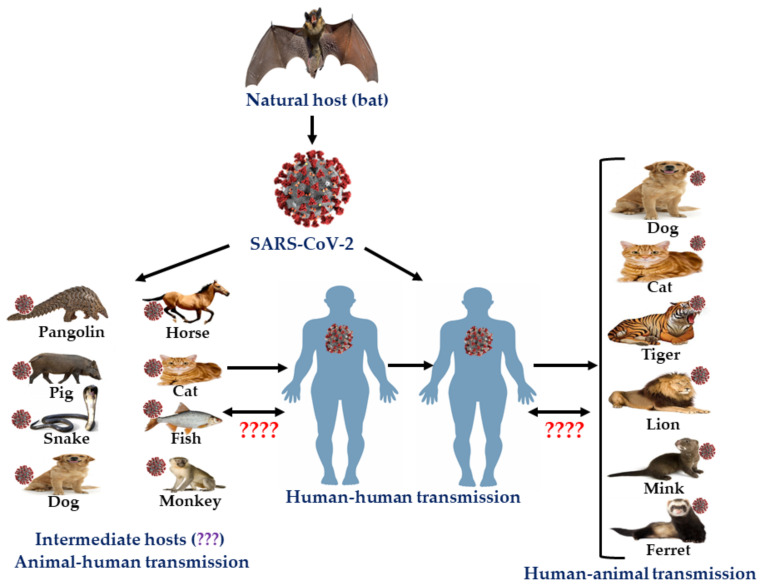
Zoonotic features of SARS-CoV-2 (modified with permission from [200]).

Coronaviruses are enveloped, positive-sense single-stranded RNA viruses of the family *Coronaviridae* [155,156]. Previously six coronaviruses (CoVs) were known to cause human infections. Recently, a newly emerged coronavirus initially known as 2019-novel coronavirus (2019-nCoV) and later designated as SARS-CoV-2, was reported from China in late December 2019 causing a disease known as COVID-19, and the outbreak has been later classified as pandemic [156,157,158,159]. All seven reported human coronaviruses are zoonotic in nature. Bat is the natural host for most coronaviruses with the exception of HCoV-OC43 and HCoV-HKU1, which have originated from rodents (Table 3). A possible route of transmission to humans is through an intermediate host that humans have contact with. As the viruses are chiefly transmitted through respiratory droplets and fomites, human to human transmission is relatively easy. Contact transmission is an important factor in tracking the spread of these viruses. Among the seven viruses, SARS-CoV, MERS-CoV, and SARS-CoV-2 were the most severe. MERS-CoV had the highest fatality rate. COVID-19 is a global public health challenge and is causing devastating effects on health, social life, and economy all over the world. The disease has been reported in more than 200 countries causing 25 million cases and 0.9 million deaths worldwide [160]. The zoonotic features of SARS-CoV-2 are presented in Figure 2.

## 9. Neglected Zoonoses

**Figure 3:**
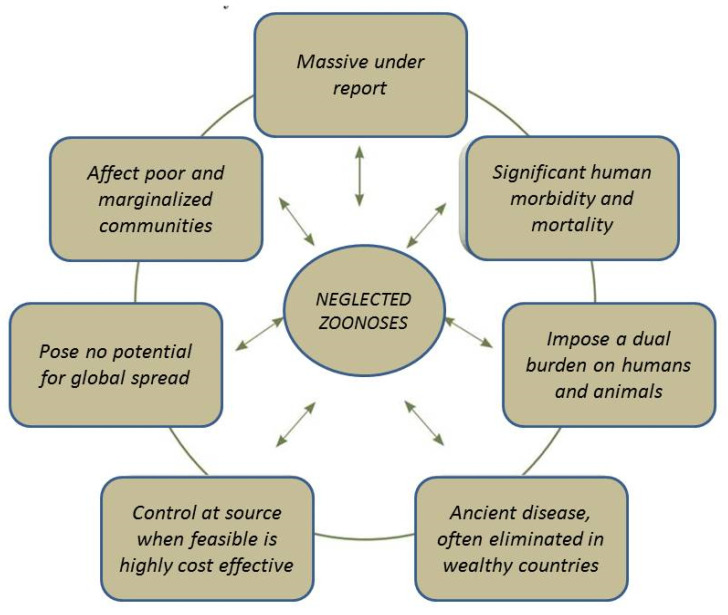
Basic features of neglected zoonotic diseases (reproduced with permission from [202]).

Many zoonotic diseases are endemic in the developing world, which negatively impacts the health conditions and livelihoods of poor people. Because of their endemic nature, they tend to be under-reported and have been largely neglected by many funding agencies compared to emerging and re-emerging zoonoses and thus have been named as neglected zoonoses [201]. Most developed countries have been successful in the control and elimination of neglected zoonotic diseases [202]. The salient features of neglected zoonoses are presented in Figure 3.

Mainly, tropical countries are more vulnerable for neglected diseases, which is why these diseases have been sometimes known as neglected tropical diseases. Since the neglected zoonotic diseases have lower priority in the health systems in many countries, they have silently triggered significant morbidity among rural people. In May 2013, delegations from 32 WHO member states made some important decisions in the “World Health Assembly” to attempt to control 17 neglected zoonotic diseases. They also implemented a WHO roadmap for the assessment of preventive and control strategies for those neglected tropical diseases [203]. Important zoonotic diseases include rabies, anthrax, cysticercosis, brucellosis, foodborne trematode infections, leishmaniasis, echinococcosis, and zoonotic sleeping sickness [204,205]. Zoonotic diseases that have been neglected (neglected zoonoses) include rabies in Africa and Asia; echinococcosis and taeniasis (*Taenia solium*) in Asia, Africa, and Latin America; leishmaniasis in Asia and Africa; and cysticercosis and foodborne trematodiasis in Africa [206].

## 10. Impact of Zoonoses

Zoonoses have myriad impacts on human and animal health. Though the impact of zoonoses is hard to quantify, it can be assessed by parameters such as disease prevalence, incidence, morbidity, mortality, and economic loss [207]. Both human livelihood and well-being are severely affected by zoonoses. The affected individuals suffer from hurdles that negatively impact their work performance and thus their ability to support their families. These situations are frequently noticed in under-developed African and Asian countries. In some cases, the affected individuals may stay isolated from the rest of the community and thus are more prone to developing mental health issues. Antibiotic resistance is a global health challenge that can negatively impact the treatment of bacterial zoonoses. Patients suffering from diseases caused by resistant bacteria need special attention, expensive medicine, and are generally a burden on the health sector, especially in developing countries.

Animal deaths caused by zoonotic diseases can impose massive economic losses on the livestock sector of any country. Even if animals do not die, animal health and productivity can still be negatively impacted. This can lead to a significant loss of animal products such as meat, milk, and eggs, which can be more than 70%. Human health and nutrition are also affected due to the reduced supply of high-protein food of animal origin such as milk, meat, and eggs [208]. Zoonotic diseases, such as brucellosis, toxoplasmosis, can lead to infertility, abortion, and weak offspring. This can cause great economic losses to farmers and to the whole country.

Zoonotic diseases such as BSE, avian influenza, and anthrax can hamper the international trade of animals and animal products (meat, milk, and eggs) and byproducts across the globe. The economy is also tremendously affected due to measures required for zoonoses control and eradication such as zoonoses surveillance, diagnosis, isolation and quarantine, restriction on animal transportation, treatment and vaccination programs, inspection of meat and milk, and biosecurity. From 1995 to 2008, the global economic impact of zoonotic outbreaks has exceeded 120 billion USD [209]. In the UK, the economic losses due to zoonotic diseases were significant [210]. In 2007, the UK faced drastic food-borne pathogen problem by *Campylobacter* spp., *Salmonella* (non-typhoidal), *E. coli* VTEC O157, *Listeria monocytogenes*, and norovirus, which caused serious economic losses [211]. In addition, other countries also faced drastic economic losses due to outbreaks of zoonotic food-borne pathogens. For example, Ireland has also suffered a severe economic loss due to *Salmonella* contamination in its pork products [211]. According to the World Bank [212], Australia has lost 16% of the value of its livestock sector due to outbreaks that impacted beef and sheep. [213].

It is noteworthy that the global economy was severely impacted by the SARS outbreak, which impacted multiple sectors including the tourism sector. The economic impact of SARS in Singapore, China, Hong Kong, and Taiwan was severe [214]. Moreover, the emergence of the highly pathogenic avian influenza significantly curtailed tourism to Mexico and resulted in economic losses to the country [215]. Likewise, India faced economic losses due to the restriction of tourism that resulted from the plague outbreak in 1994 [214]. Furthermore, Chile has suffered steep economic losses due to the outbreak of highly pathogenic avian influenza [216]. Countries in the European Union were not spared from drastic economic losses resulting from the closure of export markets of poultry during the outbreak of highly pathogenic avian influenza [211].

BSE is one of the important emerging zoonoses. When outbreaks of BSE occurred in the UK, most European countries banned importing British beef. The cost for implementing extensive control measures, including culling of all infected cattle and slaughter of at-risk animals, was exorbitant [217]. The outbreak of BSE in Toronto, Canada, resulted in the loss of 0.5% of the city’s GDP. The disease was detected in millions of animals for which many countries banned international trade with Canada [218]. Due to the detection of BSE in the U.S. in 2003, numerous countries banned the import of American beef causing drastic economic losses [219].

Brucellosis is another economically-important zoonoses. Brucellosis in cattle resulted in annual economic losses in Kenya [220], Argentina [221], and Nigeria [222]. The recent COVID-19 outbreak has significantly impacted the global economy. COVID-19 has significantly impacted all sectors of the society including health and education sectors, financial sectors, travel and hospitality sectors, and the sports sector [223]. Travel industry is poised to lose significant revenue due to the pandemic [223]. It is estimated that millions of people will face extreme poverty due to the stalled growth resulting from this pandemic [224].

The disease burdens associated with zoonoses are presented in Table 4. As revealed from the table, emerging zoonoses are mostly concentrated in more developed countries whereas endemic zoonoses are mostly located in developing countries.

## 11. Control of Zoonoses

Zoonoses present a serious health threat to the international community. About 58–61% of the human diseases are communicable and up to 75% are zoonotic (transmitted from animals) [226,227]. Zoonosis involves the interaction of humans, animals, and environment, and therefore a multi-sectorial approach is required to ensure effective control measures [228].

Surveillance is crucial to prevent and control zoonotic diseases. It can be used to detect early infection, affected humans and animals, reservoirs, vectors, and endemic areas including the “hotspots”. It helps in the adaptation of control strategies against emerging and re-emerging diseases to improve human health status, to manage disease properly, and to minimize morbidity and mortality of humans and animals. Since zoonoses (such as SARS and HPAI) can spread swiftly across the globe to affect global communities, coordinated surveillance approaches at local, regional, national, and international levels are essential to control zoonoses. All potential sources of zoonoses including exotic animals and birds, pet and companion animals, aquatic animals, wildlife, and rodents need to be subject to surveillance. There are different types of surveillance that need to be conducted [229]. Successful and functional surveillance requires well equipped lab, adequate diagnostic facilities, skilled manpower and funding. The following four surveillance types can be practiced for the control of zoonoses:

- Pathogen surveillance to detect and identify pathogens.
- Serological surveillance to detect the presence of pathogens in the blood of humans or animals through monitoring immune responses.
- Syndrome surveillance to determine the propensity of diseases through data analysis based on symptoms. This analysis-based surveillance cannot be used identify the presence of pathogens.
- Risk surveillance to detect risk factors responsible for the transmission of disease. This control strategy cannot be used to determine the clinical features of multifarious diseases along with their prevalence.

General principals of disease control such as providing treatment to affected individuals, vaccination of healthy individuals and animals, restricting animal movement, animal population control, and test and cull (anthrax, glanders, and Rift Valley fever) can also be used for the control of zoonoses. Decontamination of infected materials is needed to reduce the chances of acquiring new infections. For example, safe disposal of aborted fetus can reduce the prevalence of brucellosis. Personal hygienic management and usage of personal protective equipment such as gloves, masks, lab coats, helmets, and goggles need to be practiced. When applicable, thorough disinfection of contaminated materials and areas need to be carried out to assist in reducing the spread of brucellosis, salmonellosis, and tuberculosis.

Concerted and multidisciplinary approaches are required for the control of emerging and re-emerging zoonoses [230]. Many emerging and re-emerging infections (such as dengue fever, Zika, and chikungunya) are vector-borne arboviral infections. Therefore, for their effective control, an effective epidemiological surveillance of these infections needs to be taken under consideration in addition to vector control [231]. This surveillance includes risk factors responsible for emergence or re-emergence such as vector biology, host dynamics, pathogen niche and virulence, wildlife distribution, land use, and socio-economic status. Pest and vector control are also required to control some parasitic and bacterial zoonoses transmitted by vector-like mosquitoes, tick, and lice. Successful vector control strategies should contain a combination of physical, biological, and/or mechanical methods including integrated pest management and integrated vector management systems [232].

Many zoonoses are preventable but are neglected despite being a significant threat on public health, especially in developing countries. During zoonoses control programs, factors relating to both humans and animals need to be accounted for. Where several neighboring countries are affected, coordinated approaches need to be adopted for zoonoses control. Approaches that rely on the principles of one health policies need to be adopted and must involve veterinarians, medical doctors, occupational health physicians and public health operators, conservation officers and environmental officers for effective zoonoses control [233]. A research project named Integrated Control of Neglected Zoonoses for the control of neglected zoonotic diseases in Africa reinforced the one health-based concepts among academic and professionals from 21 European and African countries [234].

All disease control measures require a substantial amount of financial support that is usually unavailable for the developing countries. The developed countries and international donors need to support the developing countries for effective zoonoses control. Donor agencies such as WHO, Food and Agriculture Organization of the United Nations (FAO), World Organization for Animal Health (OIE), US Agency for International Development (USAID), US Department of Agriculture (USDA), European Union (EU), Department for International Development (DFID), Biotechnology and Biological Sciences Research Council (BBSRC), and Danish International Development Agency (DANIDA) could be approached for funding. Similarly, private funding bodies like the Welcome Trust and the Bill and Melinda Gates Foundation can also be approached for funding to implement zoonoses control programs [235].

In order to control food-borne zoonoses, an ample supply of safe food has to be made available for consumers. This could be achieved through implementing two broad approaches: risk assessment and risk management of food products. Risk assessment can be conducted through collecting and analyzing data, and by providing recommendations based on importance; risk management should be adopted by taking legislative measures and setting targets to reduce the risk. Animal-origin foods such as meat, milk, and eggs have to originate from healthy animals that are free of zoonotic pathogens. Proper ante-mortem and post-mortem examination of animals is vital to ensure safety of food from animal origin. Ensuring hygienic conditions in every step of food processing including personal hygiene of personnel involved in food processing need to be monitored for the production of safe food.

Other activities for the control of zoonoses include issuing laws and regulations related to the isolation and quarantine, establishment strong and effective disease reporting (notification) systems, farm biosecurity, mass vaccination, test and slaughter or cull, public awareness, and health education. Mass media and electronic information system, social networks, text messages and other communication channels can play a significant role in increasing public awareness to control zoonoses.

### Zoonoses and One Health

For the prevention and control of infectious diseases such as zoonotic diseases, international organizations and researchers described the relationship among human, animals, and environments and adopted a concept known as “One Health Concept” or “One Health Approach”. This concept was adopted to properly deal with global health challenges [225]. The one health concept encourages collaborations among wildlife biologists, veterinarians, physicians, agriculturists, ecologists, microbiologists, epidemiologists, and biomedical engineers to ensure favorable health for animals, humans, and our environment [228,236,237]. The one health approach has widespread impacts on poverty, food security, and health security through the prevention and control of zoonoses, mainly in the developing countries [238].

For the prevention and control of emerging and re-emerging diseases including zoonoses, the collaborations and partnerships of multi-sectoral personnel are badly needed for the implementation of feasible operations and surveillance among the human, animals, and environmental sectors [236]. WHO, OIE, FAO, US Centers for Disease Control and Prevention (CDC), US Department of Agriculture (USDA), United Nations System Influenza Coordination (UNSIC), and European Commission recognize the prevention and control strategies involving the one health approach [239,240,241].

One health is directly linked in the prevention and control of zoonoses. According to Pieracci et al. [242], the recommendations provided by one health approach to prevent and control zoonoses are (1) developing “Zoonotic Disease Unit” for betterment of the human and animal health agencies; (2) developing national strategy for “Zoonotic Disease Unit”; (3) engaging leadership among multi-sectoral researchers and relevant personnel to prioritize zoonotic disease research; (4) adopting veterinary public health policies with collaborators from other countries; and (5) reviewing the zoonotic diseases on a regular basis (2–5 years) to address the emerging and re-emerging diseases through regular surveillance, epidemiological implementations, and laboratory diagnosis.

Experts from CDC’s one health office have suggested some strategies in the One Health Zoonotic Disease Prioritization to prevent and control zoonotic diseases [239]. The recommendations include (1) developing a list of emerging and re-emerging zoonotic diseases based on priority by the delegated one health sectors; (2) implementing recommendations for further steps to reduce the intensification of diseases; (3) realizing the role and importance among represented one health sectors; (4) strengthening and creating the one health coordination programs; and (5) generating reports on zoonotic diseases on national, subnational, and regional levels.

In brief, the one health concept plays a significant role to address emerging and re-emerging zoonoses; to control the effect of zoonotic diseases among humans, animals, and environmental components; and to make the world free from threats of zoonotic diseases.

## 12. Recommendations

Zoonotic diseases represent a serious public health concern. Many zoonoses are currently under control but there is gap in our knowledge about many diseases especially on the disease distribution, etiology, pathogen, host, vector biology, dynamics, transmission cycle, predisposing factors, and risk factors. The equilibrium exists among the host, agent, and environment may be disturbed at any point due to various anthropogenic activities of increasing human population and natural activities to evoke emission of zoonoses. With the existing knowledge we cannot accurately predict the time or the impact of the next pandemic of a zoonoses may occur. The following activities need to be ensured or strengthened for our preparedness to overcome such pandemic.

- Active and wider zoonoses surveillance and monitoring with advanced tools like satellite-based remote sensing system and molecular epidemiological tools.
- Disease reporting and notification service.
- Giving priority to zoonoses and action team formation.
- Available diagnostic facilities and skilled manpower.
- Cooperation at regional, national, subnational, and international levels.
- One health-based approach comprising both veterinarians and medical doctors in addition to environmental experts and other professionals.
- Ensuring adequate regular and emergency funding.
- Mass campaigning on public awareness on zoonoses.
- More research on disease epidemiology, risk factors, pathogen virulence, host biology, and vector biology.
- Wildlife monitoring and wildlife protection.
- Ensure safe food production of animal origin.
- Ensure safety of infectious laboratories to avoid the accidental spread of zoonotic infections and bioterrorism.
- Protection of environment.
- National and international educational programs to make people aware of zoonoses and hygiene.

## 13. Conclusions

The majority of the human infectious diseases have animal origins. These pathogens do not only cause diseases in animals, but they also pose a serious threat for human health. In many cases the altered food habit, climate change, and environmentally unfriendly human operations influence the emergence and reemergence of many zoonotic diseases because of the increased contact between humans and wild animals. The devastating impact of zoonosis on the human population is evident from the current COVID-19 pandemic. Because of the strong interrelatedness among animals, humans, and environment; research focusing on the one health approach need to be prioritized to identify critical intervention steps in the transmission of pathogens. Robust active surveillance targeting all components of the one health approach need to be implemented to early and accurately detect zoonoses, so that effective control measures could be taken.

## Figures and Tables

**Table 1:** Major Zoonotic Diseases, their etiological agents, hosts, and the major symptoms in humans.

Disease	Etiology	Animal Host	Major Symptoms, System or Organs Involved
Bacterial zoonoses			
Anthrax	*Bacillus anthracis*	Cattle, horses, sheep, pigs, dogs, bison, elks, white-tailed deer, goats, and mink	Skin, respiratory organs, or GI tract
Tuberculosis	*Mycobacterium bovis,* *Mycobacterium caprae,* *Mycobacterium microti*	Cattle, sheep, swine, deer, wild boars, camels, and bison	Respiratory organs bone marrow
Brucellosis	*Brucella abortus* *Brucella melitensis,* *Brucella suis,* *Brucella canis,*	Cattle, goats, sheep, pigs, and dogs	Fever, usually high in the afternoon, back pain, joint pain, poor appetite, and weight loss
Bubonic plague	*Yersinia pestis*	Rock squirrels, wood rats, ground squirrels, prairie dogs, mice, voles, chipmunks, and rabbits	Fever, chills, abdominal pain, diarrhea, vomiting, and bleeding from natural opening
Glanders	*Burkholderia mallei*	Horses, donkeys, and mules	Fever, sweating, muscle aches, chest pain, muscle tightness, and headache
Leprosy	*Mycobacterium leprae*	Monkeys, rats, mice, and cats	Skin lesions
Leptospirosis	*Leptospira interrogans*	Wild and domestic animals including pet dogs	Fever, abdominal pain, jaundice, and red eye
Tularemia	*Francisella tularensis*	Rabbits, squirrels, muskrats, deer, sheep, bull snakes, wild rodents, beavers, cats, and dogs	Joint pain, diarrhea, and dry cough
*Arcobacter* infections	*Arcobacter butzleri,* *Arcobacter cryaerophilus,* *Arcobacter skirrowii*	Cattle, sheep, pigs, and chickens	Abdominal pain, fever, and vomiting
Actinomycosis	*Actinomyces bovis*	Cattle, sheep, horses, pigs, dogs, and other mammals	Swelling of lymph nodes, soft tissues, skin, and abscess
Bordetellosis	*Bordetella bronchiseptica*	Cats and dogs	Respiratory problem
Lyme disease	*Borrelia burgdorferi*	Cats, dogs, and horses	Fever, headache, skin rash, and erythema migrans
Campylobacter enteritis	*Campylobacter jejuni*, *Campylobacter coli*	Cattle, sheep, chickens, turkeys, dogs, cats, mink, ferrets, and pigs	Enteric disorder
*Campylobacter fetus* infection	*Campylobacter fetus* subsp. *fetus*, *Campylobacter fetus* subsp. *testudinum*	Cattle, sheep, and goats	Enteric disorder
*Clostridioides difficile* infection	*Clostridioides difficile*	Cattle, horses, and birds	Pseudomembranous colitis, and diarrhea
*Corynebacterium ulcerans* and *Corynebacterium pseudotuberculosis* infections	*Corynobacterium ulcerans,* *Corynobacterium pseudotuberculosis*	Cattle, dogs, and cats	Diphtheria
Enterohemorrhagic *Escherichia coli* infections	*E coli* O157:H7	Cattle, sheep, pigs, deer, dogs, and poultry	Enteritis and Hemolytic–uremic syndrome (HUS)
*Helicobacter* infection	*Helicobacter pullorum*, *Helicobacter suis*	Poultry and pigs	Peptic ulcer
Vibriosis	*Vibrio parahaemolyticus*	Farm animals	Enteritis
Salmonellosis	*Salmonella enterica,* *Salmonella bongor*	Domestic animals, birds, and dogs	Enteritis
Ehrlichiosis	*Anaplasma phagocytophilum,* *Ehrlichia ewingii,* *Ehrlichia chaffeensis,* *Ehrlichia canis,* *Neorickettsia sennetsu*	Sheep, cattle, deer, dogs, and cats	Fever, headache, fatigue, muscle aches, and occasionally rash
Pasteurellosis	*Pasteurella multocida*	Poultry, pigs, cattle, buffaloes, sheep, goats, deer, cats, dogs, and antelope	Fever, vomiting, diarrhea, and gangrene
**Viral zoonoses**			
Rabies	Rabies virus, Genus—*Lyssavirus* Family—*Rhabdoviridae*	Cattle, horses, cats, dogs, bats, monkeys, wolves, skunks, rabbits, and coyotes	Nervous system
Newcastle disease	Paramyxovirus, Genus—*Avulavirus* Family—*Paramyxoviridae*	Poultry and wild birds	Conjunctivitis
Avian influenza	Influenza A virus Genus—*Alphainfluenzavirus* Family—*Orthomyxoviridae*	Ducks, chickens, turkeys, dogs, cats, pigs, whales, horses, seals, and wild birds	Flu like symptoms, diarrhea, and pneumonia
Rift Valley fever	Rift Valley fever virus Genus—*Phlebovirus* Family—*Bunyaviridae*	Buffaloes, camels, cattle, goats, and sheep	Influenza- like fever, muscle pain, joint pain, and headache
Ebola virus disease (Ebola Hemorrhagic Fever)	Ebola virus Genus—*Ebolavirus* Family—*Flaviviridae*	Monkeys, gorillas, chimpanzees, apes, and wild antelopes	Fever, intense weakness, muscle pain, headache, sore throat, hemorrhage, vomiting, diarrhea, kidney, and liver failure
Marburg viral hemorrhagic fever	Marburg virus Genus—*Marburgvirus* Family—*Flaviviridae*	Fruit bats and monkeys	Hemorrhage, fever, muscle pains, watery diarrhea, abdominal pain, and non-itchy rash
Chikungunya fever	Chikungunya virus Genus—*Alphavirus* Family—*Togaviridae*	Monkeys, birds, and rodents	High fever, severe joint pain, muscle pain, and skin rash
Dengue fever	Dengue virus Genus—*Flavivirus* Family—*Flaviviridae*	Monkeys and dogs	High fever, skin rash, skin hemorrhage, and shock
Hantavirus infection (Hantavirus Pulmonary Syndrome)	Hantavirus Genus—*Orthohantavirus* Family—*Hantaviridae*	Deer mice, cotton rats, rice rats, white-footed mice, shrews, and moles	Respiratory problem, high fever, dizziness, chills, and abdominal problems
Zika fever	Zika virus Genus—*Flavivirus* Family—*Flaviviridae*	Apes and monkeys	Fever, pain, and conjunctivitis
West Nile fever	West Nile virus Genus—*Flavivirus* Family—*Flaviviridae*	Horses, birds, and reptiles	Headache, skin rash, swollen lymph nodes, stiff neck, disorientation, coma, tremors, convulsions, and paralysis
AIDS	HIV Genus—*Lentivirus* Family—*Retroviridae*	Monkeys and chimpanzees	Immunosuppression, influenza-like symptoms, fever, chills, rash, night sweats, muscle aches, fatigue, swollen lymph nodes
Severe acute respiratory syndrome (SARS)	SARS coronavirus (SARS-CoV) Genus—*Coronavirus* Family—*Coronaviridae*	Bats, dogs, cats, ferrets, minks, tigers, and lions	influenza-like symptoms, fever, muscle pain, severe cases progress to a respiratory disease and pneumonia
Monkey pox	Monkeypox virus Genus—*Orthopoxvirus* Family—*Poxviridae*	Squirrels, Gambian poached rats, dormice, different species of monkeys, and others.	Fever, pox lesions on skin
**Parasitic zoonoses**			
Trichinellosis	*Trichinella* spp.	Pigs, dogs, cats, rats, and other wild species	Gastrointestinal, e.g., nausea, vomiting, diarrhea, and abdominal pain
Visceral larva migrans	*Baylisascaris procyonis*, *Toxocara canis*, *Toxocara cati*, and *Ascaris suum*	Birds, emus, cats, chinchillas, porcupines, prairie dogs, rabbits, weasels, woodchucks, and woodrats	Gastrointestinal, e.g., coughing, shortness of breath, fever, and abdominal pain
Cutaneous larval migrans	*Ancylostoma braziliense*	Dogs and cats	Subcutaneous tissue
Hydatidosis	*Echinococcus granulosus*	Buffaloes, sheep, goats and adult stray or shepherd dogs	Hydatid cysts in liver, lungs, bones, kidneys, spleen, abdominal pain, and respiratory problem
Cryptococcosis	*Cryptococcus neoformans*	Dogs, cattle, horses, sheep, goats, birds, and wild animals	Respiratory problems, fever, nausea, and vomiting
Cryptosporidiosis	*Cryptosporidium parvum*	Cattle, sheep, pigs, goats, horses, and deer	Diarrhea lasting 3–14 days. Abdominal pain, nausea and malaise are frequent. Some patients have a slight fever
Fascioliasis	*Fasciola hepatica,* *Fasciola gigantica*	Cattle, sheep, goats, and other ruminants	Intense internal bleeding, fever, nausea, swollen liver, skin rashes, and extreme abdominal pain
**Mycotic/Fungal zoonoses**			
Tinea/ringworm infection	*Microsporum* spp., *Trichophyton* spp.	All animals like cattle, sheep, goats, cats, and dogs	Skin lesions
Aspergillosis	*Aspergillus* spp.	All domestic animals and birds	Respiratory problems
Blastomycosis	*Blastomyces dermatitidis*	Mostly dogs, cats, and less common in horses, ferrets, deer, wolves, African lions, bottle-nosed dolphins, and sea lions	Fever, malaise, pneumonia, verrucous skin lesions, subacute meningitis, gait abnormalities, and seizures
Coccidioidomycosis	*Coccidioides immitis*, *Coccidioides posadasii*	Dogs, horses, pigs, and ruminants	Hypersensitivity reaction, fever, erythema nodosum, erythema multiform, arthralgia, pleuritic chest pain, and dry cough
Cryptococcosis	*Cryptococcus neoformis*	Cats, dogs, cattle, horses, sheep, goats, birds, and wild animals	Meningitis, fever, malaise, headache, neck stiffness, photophobia, cough, nausea, and vomiting
Sporotrichosis	*Sporothrix schenckii*	Dogs, cats, horses, cows, camels, dolphins, goats, mules, birds, pigs, rats, and armadillos	Erythematous papulonodular lesions, cough, low-grade fever, weight loss, pulmonary dysfunction, and lung abscess
Malassezia infection	*Malassezia* spp.	Dogs and cats	Pityriasis versicolor, seborrheic dermatitis, atopic eczema, folliculitis, and dandruff
Histoplasmosis	*Histoplasma capsulatum* var. *capsulatum*	Cats, dogs, rabbits, and rats	Often asymptomatic, fever, productive cough, chest pain, weight loss, hepatosplenomegaly, and hematologic disturbances
**Rickettsial zoonoses**			
Q-Fever	*Coxiella burnetti*	Cattle, sheep, goats, dogs, cats, chickens, and wild animals	Fever, and skin rash
Epidemic typhus	*Rickettsia prowazekii*	Dogs, lambs, goat kids, calves, donkeys, and young camels	High fever, headache, malaise, myalgia, arthralgias, rashes, CNS manifestations, petechiae, and cough
Rocky mountain spotted fever	*Rickettsia rickettsii*	Rodents and dogs	Fever, headache, rash, malaise, myalgia, anorexia, nausea, vomiting, abdominal pain, and photophobia
Queensland tick typhus	*Rickettsia australis*	Bandicoots, rodents, cattle, wombats, and companion animals	Mild fever, macular, papular, or maculo-papular rash, rigors, myalgia, arthralgia, acute renal failure, headache, and lymphadenopathy
Scrub typhus	*Orientia tsutsugamushi*	Rodents	Fever, rash, myalgia, diffuses lymphadenopathy, necrotic eschar, cough, and headache, diarrhea
**Chlamydial zoonoses**			
Enzootic abortion	*Chlamydia abortus*	Cattle, horses, sheep, pigs, cats, and rabbits	Abortion
Psittacosis	*Chlamydia psittaci*	Parrots, parakeets, lories, cockatoos, cattle, sheep, and goats	Cough, dyspnea, pleuritic chest pain, epistaxis, sore throat, hemoptysis, fever, malaise, anorexia, chills, nausea, vomiting, myalgias, arthralgias, headache, and abdominal pain
Chlamydiosis	*Chlamydia felis,* *Chlamydia trachomatis*	Cats and mice	Conjunctivitis, urethritis, cervicitis, pelvic inflammatory disease, ectopic pregnancy, tubal factor infertility, epididymitis, proctitis, and reactive arthritis (sequelae)
**Protozoal zoonoses**			
Trypanosomiasis	*Trypanosoma brucei*	Antelopes, cattle, camels, and horses	chronic and intermittent fever, headache, pruritus, lymphadenopathy, hepatosplenomegaly, and sleep disturbance
Leishmaniasis	*Leishmania infantum*	Cats, dogs, horses, and bats	Skin lesions, hepatosplenomegaly, and wasting
African sleeping sickness	*Trypanosoma brucei*	Antelopes, cattle, camels, and horses	High fever, headache, nausea, vomiting, and erythematous plaque formation
Chagas disease	*Trypanosoma cruzi*	Domestic pigs and cats, wildlife reservoirs include opossums, armadillos, raccoons, and woodrats	severe myocarditis, meningoencephalitis, swelling or redness of skin, fever, swollen lymph nodes, head or body aches, fatigue, nausea, vomiting, and diarrhea
Giardiasis	*Giardia lamblia*	Dogs, cats, ruminants, and pigs	Diarrhea, abdominal cramping, bloating, flatulence, malaise, nausea, and anorexia
Toxocariasis	*Toxocara canis,* *Toxocara cati*	Dogs and cats	Fever, anorexia, hepatosplenomegaly, rash, pneumonitis, asthma, and visual impairment
Toxoplasmosis	*Toxoplasma gondii*	Pigs, sheep, goats, poultry, and rabbits	Lymphadenopathy, fever, malaise, night sweats, myalgia, sore throat, and maculopapular rash
Balantidiasis	*Balantidium coli*	Ruminants, pigs, guinea pigs and rats	Chronic diarrhea, occasional dysentery, nausea, foul breath, colitis, abdominal pain, weight loss, and deep intestinal ulcerations
**Disease caused by acellular non-viral pathogenic agents**			
Mad Cow Disease, also known as BSE (Bovine spongiform encephalopathy). In human known as Creutzfeldt–Jakob disease (CJD)	Prion protein	Cattle, sheep, goats, mink, deer, and elks	Ataxia, jerky movements, seizures, dementia, memory loss, and personality changes

**Table 2:** Examples of reverse zoonoses (retrieved from [21]).

Agent	Human Disease	Animal Disease	Animal Affected
Mumps virus	Mumps	Parotiditis	Dogs
Infectious hepatitis	Hepatitis	Hepatitis	Nonhuman primates
*Corynebacterium diphtheriae*	Diphtheria	Ulcers on teats, mastitis	Cattle
*Staphylococcus aureus*	Furunculosis	Furunculosis, mastitis	Cattle
*Streptococcus pyogenes*	Pharyngitis, scarlet fever	Mastitis	Cattle
*Giardia lamblia*	Nausea, flatulence diarrhea	None known	Beavers
*Mycobacterium tuberculosis*	Tuberculosis	Tuberculosis	Deer, dogs, elephants

**Table 3:** Salient features of different zoonotic coronaviruses.

Virus	HCoV-229E	HCoV-NL63	HCoV-OC43	HCoV-HKU1	SARS-CoV	MERS-CoV	SARS-CoV-2
Genus	*Alphacoronavirus*		*Betacoronavirus*				
Disease	Mild respiratory tract infections			Mild respiratory tract infections and pneumonia	Severe acute respiratory syndrome	Middle East respiratory syndrome	Coronavirus disease 2019
Natural host	Bats		Rodents		Bats		
Intermediate host	Camelids?	Unidentified	Bovines	Unidentified	Palm civets	Dromedary camels	Unidentified
Transmission	Respiratory droplets, aerosols, and fomites				Respiratory droplets, aerosols, fomites and fecal–oral	Respiratory droplets and fomites	Respiratory droplets, aerosols, fomites, and fecal–oral (?)
Incubation period (days)	2–5	2–4	2–5	2–4	2–11	2–13	1–14
Signs and symptoms	Fever, nasal discharge, sneezing, cough, malaise headache, and sore throat	Fever, dry cough, headache, myalgia, malaise, dyspnea, respiratory distress, and diarrhea	Fever, nasal discharge, sneezing, cough, malaise headache, and sore throat	Fever, cough, running nose, and dyspnea	Fever, headache, dry cough, dyspnea, respiratory distress, malaise, myalgia, and diarrhea	Fever, cough, myalgia, chills, sore throat, dyspnea, pneumonia, arthralgia, diarrhea, vomiting, and acute renal impairment	Fever, dry cough, headache, dyspnea, myalgia, respiratory distress, renal impairment, diarrhea, multiple organ failure
Case fatality	N/A	N/A	N/A	N/A	9.6%	34.4%	3.5%
Epidemiology	Peak in winter globally				2002–03 in China, then Globally to 29 countries	2012 in Middle East, 2015 in South Korea, and Endemic in Middle East	2019–2020 in China, then progressed to a global pandemic.
References	[161,162,163,164,165,166,167]	[168,169,170,171,172,173]	[165,174]	[174,175]	[176,177,178,179,180,181,182,183]	[184,185,186,187,188,189,190,191,192,193,194,195,196]	[155,197,198,199]

**Table 4:** Disease burdens associated with zoonoses (retrieved from [6,225]).

Poverty Interface		Emerging Market Interface		Zoonoses Interface		
Poor livestock Keepers	Protein Energy Malnutrition	Monogastrics (TLU) 2010	Rapid Change Monogastrics 2010–2030	Zoonoses Burden (GBD)	Endemic Zoonoses Prevalence	Emerging Zoonoses Events
Bangladesh	Bangladesh	Bangladesh	Myanmar	India	Nigeria	USA
India	India	India	Burkina Faso	Nigeria	Ethiopia	UK
Pakistan	Pakistan	Pakistan	India	Congo DR	Tanzania	Australia
China	China	Myanmar	Pakistan	China	Togo	France
Kenya	Ethiopia	China	Ghana	Ethiopia	India	Brazil
Nigeria	Nigeria	Thailand	Afghanistan	Bangladesh	Mali	Canada
Sudan	Indonesia	Indonesia	Bangladesh	Pakistan	Vietnam	Germany
Congo DR	Congo DR	Vietnam	Liberia	Afghanistan	Sudan	Japan
Ethiopia	Angola	Iran	Central African Republic	Angola	Bangladesh	China
			Chad			
			Cambodia			
Tanzania	Afghanistan	Philippines	Benin	Brazil	Burkina Faso	Sweden
Turkey	Sudan	Brazil	Laos	Indonesia	Cameroon	Italy
Indonesia	Philippines	Nigeria	Thailand	Niger	Chad	Malaysia
Niger	Brazil	Peru	Zimbabwe	Tanzania	Rwanda	Switzerland
Uganda	Uganda	South Africa		Kenya	Ghana	Congo DR
Madagascar	Mali	Morocco	Ethiopia	Côte d′Ivoire	Mozambique	Sudan
			Guinea	Uganda		
Philippines	South Africa	Ecuador	Guinea-Bissau	Sudan	South Africa	Argentina
Afghanistan	Vietnam	Colombia	China	Burkina Faso	Congo DR	India
Egypt	Tanzania	Ukraine	Mali	Mali	Egypt	Israel
Mozambique	Mozambique	Bolivia		Iraq	Gambia	Peru
Burkina Faso	Malawi	Egypt			Ivory Coast	Trinidad and Tobago
						Uganda
					Pakistan	Vietnam
					Zimbabwe	

Here, TLU = Tropical Livestock Units; GBD = Global Burden of Disease.
